# Treatment of Wounds or Surgical Dressings

**Published:** 1911-01

**Authors:** L. Sexton

**Affiliations:** Tulane, New Orleans


For Texas Medical Journal.

### Treatment of Wounds or Surgical Dressings.

BY DR. L. SEXTON, TULANE, NEW ORLEANS.

A wound is a sudden solution of the continuity of the soft parts. There are fifteen adjectives describing wounds, which we will not burden you to try to remember, but many of these descriptions have a bearing on the treatment of the injury.

Immediately following this sudden solution of continuity of tissue we have discoloration, pain, swelling and hemorrhage.

The element of pain incident to such an injury depends largely upon the location upon which the wound has been received and the character of the implement with which it was produced. For instance, a steel-jacketed bullet with high velocity may pass through the body unnoticed, the wound being discovered later by the blood trickling down from its opening. Incised wounds made by sharp instruments, as surgeons' knives, cause very much less pain than do lacerated and contused wounds. Wounds of nerve trunks of the testicle, of the elbow, and abdomen, and of the hand and finger are characterized by severe pain. A wound in inflamed tissue, as in lancing an abscess, is always attended with great pain. Increased tension and pressure upon the sensory nerve filaments is the explanation of these painful injuries.

In all wounds there is a discharge of fibrin, lymph and serum from the vessels, which must be absorbed by some superimposed sterilized gauze dressing. The arrest of hemorrhage and removal of foreign bodies is an important consideration in the first-aid treatment to injured soft parts. If an artery is severed which is too large in caliber to be arrested by torsion, it should be picked up, separated and ligated. Very often if such vessels are compressed by artery forceps for a short time, they stop bleeding without other treatment. If the wound is just above some bony prominence, direct pressure will often arrest the hemorrhage better than the insertion of sutures as ligatures. Continuous oozing from the wound (as in a large amputation) may be controlled by flushing with hot sterile solution, or by fanning the surface briskly while it is exposed to the air.

A suture often acts as a ligature in arresting hemorrhage; this is particularly true if the suture is of the button-hole variety. It should be remembered, however, that we often strangulate tissue by tying our sutures too tight in trying to arrest hemorrhage by this method. Adrenalin chloride 1 to 1000 on pledgets of cotton is a useful hemostat. It may be injected into cavities, as the urethra, or sprayed into the nostrils to arrest bleeding. In venus oozing, elevation and direct pressure are effectual in many cases; if from an artery, the tourniquet will act better.

It is embarrassing to have to open up a wound as in the scrotum to turn out blood clots and catch up oozing vessels at the second dressing which should have been attended to at the first.

All chemical and constitutional remedies for the arrest of hemorrhage are obsolete in wounds newly made, but are admissible in hemorrhage from the relaxed uterus and from ulcerated cancerous conditions.

Any of the numerous different methods of arresting hemorrhage mentioned in lectures or text-books may be utilized in the first-aid treatment of wounds.

The next most important step in the management of wounds, after the hemorrhage has been stopped, is to cleanse them. It depends largely upon the location of the wound as to what method of cleansing should be adopted; if, for instance, under the clothing on a clean skin surface partly covered with hair, the adjacent tissues should be shaved to prevent the hair from infecting the wound and keeping its edges separated. If the wound is on the scalp, it is best to shave the proximate skin to lift up the edges of the flap in order to remove any foreign body which might have been forced under the scalp at the time of the accident. While this is the general procedure, it might be occasionally modified for cosmetic purposes, if the wound happened to be on the scalp of a female, then under such circumstances strands of hair on either side of the wound might be tied across in order to approximate the edges. I have no personal experience with this method.

In lacerated or crushed injuries, use hot sterile or saline flushing to wash out foreign particles. A great many surgeons, however, prefer a 50 per cent solution of peroxide of hydrogen warmed and poured into the wound. The oxidation with the blood boils out many small particles, carrying infection which might not otherwise be detected. As a matter of course, any splinters or other foreign bodies, such as iron filings, etc., should be carefully removed either with forceps or a magnet. Blood clots and tissue with the life crushed out had as well be removed at the first dressing, when the parts are usually benumbed, as to wait for decomposition and sloughing before removing them. We may safely assume that most accidental wounds are infected, hence the importance of thoroughly cleaning them out at this first dressing; stitches should be loosely applied and drainage in the most dependent portion of the wound provided for, as it is not good surgery to hermetically seal accidental wounds either with collodion or adhesive plaster, unless material for absorption of wound secretions are first provided for. If nerves or tendons have been severed, they should be approximated by sutures whenever it is possible to find the severed ends. If the wound is very extensive, and there is likelihood of foreign bodies being driven into sensitive parts, it sometimes becomes necessary to cocainize or in the severe cases to anesthetize the patient in order to thoroughly cleanse and approximate the parts at the first dressing.

In accidents from toy pistols and giant fire crackers, garden rakes, or in injuries inflicted around dairies or barns, and in any severe laceration and contusion of the extremities, it is considered necessary to give 20 c.c. of anti-tetanic serum as a prophylaxis against tetanus.

In very delicate skin that has been protected by clothing in children and in females, the rough brush scrubbing act has been overdone; in fact, this excessive scrubbing of injuries incident to machinists, miners, railroad men, etc., with oil, grease and iron ore ingrained into the tissue, it is almost a physical impossibility to get rid of this grime at the first dressing either by scrubbing or any other process.

A great many such cases are now dressed by saturating a pledget of cotton with spirits of turpentine and rubbing off much of the grease and paint as the turpentine will dissolve; then applying a dilute tincture of iodine into the wound after it has been thoroughly flushed with any of the mild antiseptic lotions.

Such cases kept dressed several days with a moist half per cent carbolic solution, or 1 to 100,000 bichloride, will prevent infection in the wound, and so soften up these calloused hands that what was impossible to wash off at the first dressing is easily scraped off after forty-eight or seventy-two hours' application of these moist antiseptic solutions. In all deep wounds involving large areas or injury to the bone, *drainage* is advisable, at least for the first twenty-four or forty-eight hours. The ordinary cigarette drain, sterilize gauze rubber tubing or ordinary rubber tissue may be used according to the individual surgeon's experience. Only one certain rule should be observed; i. e., the drainage must be in the most dependent portion even if this requires a counter-opening to get it. Dead spaces impossible of obliteration require drainage as do amputations of the breast and thigh and suppurative appendectomies. It should be remembered, however, that all drainage is predisposed to hernia of the underlying viscera.

In wounds in the pleural cavity to evacuate pus, free drainage with double rubber tubing, and in most cases the resection of a rib becomes necessary in order that the drainage may be complete. In unhygienic surroundings with untrained help to rely upon, we should hesitate to close any lacerated or contused wound; under more favorable surroundings and with our individual inspection, it is best to close many of them, using drainage in the cases which we would otherwise treat openly. Punctured, butcher dissecting wounds should be very rarely closed, and wounds in the palm of the hand and soles of the feet are best treated openly; those in vascular tissue where the skin is loose are perhaps better stitched with silkworm cut. It has been found best to hermetically seal without drainage gunshot wounds produced by the steel-jacketed bullet, in our recent wars. Whenever it is decided to close a wound, the interruped silkworm gut suture is preferable on account of being non-irritating, less likely to infection and absorbing no moisture. The horse hair is used with equal success in closing many external surgical wounds. Some surgeons prefer the continuous, others the Michel clamp, while some use the sub-cuticular stitch for cosmetic purposes. If the Z. O. plaster is used to approximate wounds, small fenesters should be cut into the plaster just above the wound to provide for the escape of the oozing or wound secretion, which always follows. The collodion dressing is applicable to superficial wounds about the face where not much oozing is expected. Wounds well sewed are half healed. Tight sutures constrict and produce necrotic tissue. You can hardly tie a suture so slack that the subsequent swelling will not tighten it; the elasticity in the horse hair suture causes it to yield when swelling takes place, and it is on this account that it should be more generally used than it is. Fine silk and silkworm cut are usually preferable to the catgut where infection is feared. Any traumatic injury treated by the open method should be covered with 1-10,000 bichloride or some other antiseptic dressing. If the injury is to the leg or foot, the patient had better be confined to bed to secure rest, the hand or foot placed upon a Kelly pad and kept moist by applications every three or four hours, or by the continuous hot 1 to 10,000 bichloride solution; if the wound is not infected, on sensitive portions of the skin or on children the solution should be further diluted or weak iodine mixture or boracic acid be substituted for the bichloride.

Physiological rest is important in the treatment of all wounds. Splints to the hand and immovable dressings to the joints are great time-savers in the healing of wounds. Perfect rest means less pain and reaction and quick repair.

Whenever large raw surfaces are to be treated for a long time, it may become necessary to change to the boracic acid rather than the bichloride or carbolic. After these large raw surfaces are in proper condition, skin grafting hurries healing and prevents scar tissue. Theirsch or any method the surgeon is accustomed to be may used; usually we inspect or remove the dressing from skin grafting too early after lifting up the graft with the dressing. In large incised wounds it is just as essential to suture the muscles as it is the tendons and nerves; it may be difficult at times to find the proximal end of the tendon; the wound should be enlarged until it can be found and mended.

CONTUSED WOUNDS.

Most lacerated contused wounds are better packed and left to granulate than to suture them; if, however, they are sutured, a drain should first be put in the sutures, placed far apart and loosely tied. Elevation and rest must not be overlooked in the treatment of contused wounds, as it lessens pain and promotes healing.

Lacerated wounds are an exaggeration of the contused wounds; only shock is greater and hemorrhage and pain less.

If an extremity is literally amputated traumatically, it is usually better to secure the blood-vessels so that there can be no more hemorrhage; treat the shock by warmth and stimulation and leave the amputation for twenty-four or forty-eight hours, until reaction has set in. If the leg or arm are only hanging by skin or tendonous attachments, where a scissors amputation will remove the limb, this should be done, but any extensive surgery had best be postponed until reaction takes place; otherwise you may get the credit of having produced a surgical death when waiting might have saved the patient.

We should always remember that hemorrhage must first be controlled before any waiting is resorted to, for hemorrhage is the most common cause of shock; however, nerve blocking with cocaine permits amputation, which we otherwise would not attempt. Punctured wounds should not be probed, if they have opened the peritoneal cavity. A laparotomy should be performed and any injured viscera mended, otherwise you may have a death from shock, sepsis or hemorrhage.

In punctured wounds from blank cartridges or from garden rakes, or from barn splinters, it is best that the parts be thoroughly cocainized or the patient given a general anesthetic, opening up the wound by a crucial incision, removing the foreign bodies, wads or portion of cloth driven into the soft tissue, dropping in dilute tincture of iodine, packing with iodoform gauze, leaving the wound to heal by granulations from the bottom. As said before, all such cases should have the additional protection from tetanus by the injection of 500 c.c. of anti-tetanic serum.

### GUNSHOT WOUNDS.

Such wounds are usually treated without probing and by the local application of 1 to 10,000 bichloride; bullets may become encysted and do very little harm.

Prevention of infection is far more important than the removing of the bullet, which can be located by the X-ray and removed at any future date, provided it gives any serious trouble. Mayor Gaynor is carrying his encysted bullet, no attempt having been made to remove it.

Direct penetrating wounds of the abdomen with the lead bullet demands an immediate laparotomy, provided a competent surgeon is available. Not so much with the view of locating and removing the bullet as to mending the tear in the viscera and checking the hemorrhage. Bullet wounds in the thorax and in the extremities are usually treated expectantly without operation unless infection takes place. If a bullet passes through the chest and lodges in the spinal column, causing pressure upon the cord, immediate extraction should be performed, provided the bullet can be located by the X-ray.

All violently infected wounds of extremities should be put at rest at once, patient confined to bed, bowels thoroughly opened and large antiseptic dressing applied which are kept constantly moist either by pouring on or leaving such members under an antiseptic drip. The less handling the better. After forty-eight hours of such continuous treatment you will often find the inflammation subsiding or a local abscess formed. Rest in septic wounds is more important than in any other variety of injuries.

CONCLUSIONS.

Unless wounds are suppurating very freely, as a general proposition, they are dressed too often.

Peroxide of hydrogen injected into cavities and sinuses often carry the infection further into uninvaded tissue. Peroxide is also too strong to apply pure to newly healed tissue. Sterilized gauze without dusting powder is sufficient protection for any clean surgical wound.

Sterile water, saline solution, or very mild antiseptic solution, should always be given preference over the stronger antiseptics, which in destroying the pus cocci, at the same time destroy the new epithelial tissue by which granulating wounds are covered.

There is no better protection against infection than the free application of large sterilized pads or dressings with which they should be abundantly covered.

Absolute physiological rest by a properly applied splint or confinement in bed is a great time saver in the healing of wounds.

Silkworm sutures are much less likely to produce stitch abscesses and should be given the preference over catgut wherever practicable.

Z. O. plaster has a wide field of usefulness as a surgical appliance than has been given to it.

In redressing wounds, all materials should be thoroughly softened by warm sterile water before the dressing is removed.

